# Multidrug-resistant *Pseudomonas aeruginosa* in immunocompromised cancer patients: epidemiology, antimicrobial resistance, and virulence factors

**DOI:** 10.1186/s12879-025-11182-0

**Published:** 2025-06-11

**Authors:** Asmaa Abdulhak, Hamdallah H. Zedan, Hadir A. El-Mahallawy, Ahmed A. Sayed, Hend O. Mohamed, Mai M. Zafer

**Affiliations:** 1 Department of Microbiology and Immunology, Faculty of Pharmacy, Ahram Canadian University, Giza, Egypt; 2 Department of Microbiology and Immunology, Faculty of Pharmacy, Cairo University, Cairo, Egypt; 3 Department of Clinical Pathology, National Cancer Institute, Cairo University, Cairo, Egypt; 4 Genomics Program, Children’s cancer hospital Egypt 57357, Cairo, Egypt; 5 Department of Biochemistry, Faculty of Science, Ain Shams University, Cairo, Egypt; 6 Biological Control Research Department, Plant Protection Research Institute, Agricultural Research Center, Giza, Egypt

**Keywords:** Antimicrobial resistance, *Pseudomonas aeruginosa*, Virulence factors, Immunocompromised patients, *Bla*_NDM-1_, Ceftolozane/tazobactam

## Abstract

This study explores the antimicrobial resistance patterns and virulence properties of *Pseudomonas aeruginosa* obtained from blood samples of febrile immunocompromised patients. Fifty-two clinical isolates were examined for demographic and clinical characteristics, antimicrobial resistance profiles, and different virulence factors. Antimicrobial susceptibility testing and qualitative detection of efflux pump activity, biofilm formation, pigment production, and swarming motility were evaluated. The pathogenic potential of the isolates was assessed using the *Galleria mellonella* infection model. All isolates exhibited MDR, XDR, and PDR phenotypes. The crude 30-day mortality rate was 26.9%, with higher mortality detected in infections due to XDR strains. Colistin, aztreonam, and ceftolozane/tazobactam demonstrated the best susceptibility rates. Metallo-β-lactamase _*NDM−1*_ was the most frequently detected gene in our isolates. Efflux pump activity was significantly associated with higher MICs. Biofilm formation was predominant, with moderate to strong biofilm formers showing reduced antibiotic susceptibility. Swarming motility is associated with urinary tract infections. The *Galleria mellonella* model demonstrated higher lethality for isolates originating from respiratory tract infections. This study highlights the escalating load of antimicrobial resistance and significant virulence of *P. aeruginosa* which directly affects the available treatment strategies. The use of the relatively novel ceftolozane/tazobactam combination is threatened due to the high levels of *bla*_NDM−1_ producers detected. Colistin and Aztreonam are still offered treatment options.

**Clinical trial number**: Not applicable.

## Introduction

*Pseudomonas aeruginosa* is one of the most frequent opportunistic pathogens causing healthcare-associated infections particularly among immunocompromised hospitalized cancer patients [1]. *P. aeruginosa* blood stream infections (BSIs) were associated with disproportionately high morbidity and mortality rates compared to BSIs caused by other Gram-negative bacteria [2]. Over the past decade, the reported mortality rates for *P. aeruginosa* BSIs vary widely, ranging from 1.38 to 37.3% globally, and reaching 14.5–50% among pediatric populations [3, 4]. According to estimates from the Institute for Health Metrics and Evaluation (IHME), Egypt reported approximately 4.97 deaths per 100,000 population caused by *P. aeruginosa* in 2019, of which 1.34 deaths per 100,000 were attributed exclusively to BSIs [5].

The treatment of *P. aeruginosa* BSIs remains challenging due to its remarkable inherent and acquired resistance to multiple antimicrobials. This resistance combined with versatile virulence factors include biofilm formation, secretion of toxins and proteases, and the production of secondary metabolites that contribute to immune evasion and tissue damage [6–9].

The composite architecture of *P. aeruginosa* biofilms serve as protective niches that not only hinder antibiotic penetration but also facilitate chronic infection persistence and immune resistance [10]. Pyocyanin production initiates tissue injury through the production of reactive oxygen species leading to host tissue injury [11] meanwhile, pyoverdine production acts as a siderophore that plays a critical role in acquiring iron. Hemolysin produced by *P. aeruginosa* lyses numerous cell types and accelerates the spread of infections by facilitating tissue invasion [9].

While antibiotic resistance mechanisms in *P. aeruginosa* have been greatly studied, the interplay between resistance profiles and virulence factor expression remains inadequately described and is subject to conflicting outcomes across different geographic regions [8]. This is particularly serious in the context of immunocompromised hospitalized cancer patients, whose impaired immunity renders them highly vulnerable to invasive *P. aeruginosa* infections, yet studies investigating these relationships in such populations are limited, particularly in low- and middle-income countries like Egypt. Furthermore, few studies have addressed the local prevalence of virulence factors among clinical isolates and their correlation with antimicrobial resistance and clinical outcomes [12].

This study aims to evaluate the prevalence and antibiotic resistance profiles of *P. aeruginosa* isolated from bloodstream infections among high-risk immunocompromised cancer patients in a single Egyptian tertiary cancer hospital. In addition, we examine the relationship between these key virulence factors and resistance patterns to better comprehend the pathogenic potential of our collection. Through full microbiological characterization, this study seeks to contribute to local epidemiological data, inform infection control policies, and guide empirical treatment choices in this susceptible patient population.

## Materials and methods

### Bacterial strains

#### Collection and identification of clinical isolates

A total of 52 *P. aeruginosa* clinical isolates were collected prospectively from hospitalized cancer patients at the National Cancer Institute (NCI), Cairo University, Egypt. All the isolates were derived from blood samples of cancer patients with different types of cancer. Samples were collected during the period between January 2021 to December 2022.

Samples were initially identified phenotypically using standard microbiological techniques [8]. Identification was then confirmed using the BD PHOENIX Automated Microbiology System (Becton-Dickinson Diagnostic Systems, Sparks, MD, USA). *E. coli* ATCC 25,922 was used as the control strain for identification.

#### Clinical data collection and inclusion criteria

The patients included in the study were hospitalized cancer patients who developed bloodstream infections due to *P. aeruginosa*, confirmed by positive blood culture results. Patients with polymicrobial bacteremia or incomplete clinical data were excluded. Clinical data of the patients including hospital ward, age, sex, diagnosis, neutropenic status, clinical documented infections (CDI) of *P. aeruginosa* before blood (the primary source of infection before bloodstream), previous antibiotic intake within the last month, and the patients’ outcome after 30 days of hospitalization were documented.

#### Antimicrobial susceptibility testing

The Kirby-Bauer disc diffusion method was used to study the antimicrobial susceptibility of eight antimicrobial agents: piperacillin/ tazobactam (P/T), ceftazidime (CAZ), cefepime (FEP), aztreonam (ATM), meropenem (MEM), ciprofloxacin (CIP), amikacin (AMK), gentamicin (GEN). The results were interpreted according to the recommendations of the European Committee on Antimicrobial Susceptibility Testing (EUCAST) [13]. As the EUCAST 2022 guidelines do not provide specific breakpoints for gentamicin for *P. aeruginosa*, the disc content and the interpretive criteria were obtained using breakpoints and interpretive criteria for Enterobacterales which is the same as the epidemiological cut-off value stated by the EUCAST for the reference strain *P.aruginosa* ATCC27853.

*P. aeruginosa* isolates were classified according to Magiorakos et al. (2012) and Coyne et al. (2022) into: (i) Multidrug resistant (MDR) (ii) Extensively drug resistant (XDR) (iii) Difficult to treat resistance (DTR), and (iv) Pan drug resistant (PDR) [14, 15].

#### Determination of MIC

The minimum inhibitory concentration (MIC) of colistin, imipenem, and levofloxacin was determined using broth microdilution method following the EUCAST guidelines [13]. Ceftolozane/tazobactam MIC was determined using E-Test (Liofilchem, Roseto degli Abruzzi, Italy). The results were interpreted according to the recommendations of the EUCAST [13].

#### Phenotypic detection of metallo-beta-lactamases MβL & carbapenemase resistance genes

The phenotypic detection of MβL was conducted using the combined disc test according to Rameshkumar et al. (2022) [16]. Carbapenem-resistant K.N.I.V.O immunochromatographic test system Detection K-Set (Beijing Gold Mountain River Tech Development Co, China) was used to qualitatively detect carbapenemases (*bla*_*KPC-like*_, *bla*_*NDM- like*_, *bla*_*IMP- like*_, *bla*_*VIM- like*_, *and bla*_*OXA-48- like*_) in bacterial colonies. The test was carried out following the manufacturers’ procedures [17].

### Detection of efflux pump activity

The ethidium bromide (EtBr) cartwheel test was used to assess the efflux pump activity of the studied isolates. In this method, Tryptic soy agar (TSA) plates (HiMedia, India) were prepared containing different concentrations of EtBr (0 mg/L, 0.5 mg/L, 1 mg/L, 1.5 mg/L, 2 mg/L, and 2.5 mg/L) on the same day of the experiment. Each isolate was streaked on the EtBr plates at a concentration of 10^6^ cfu/mL in a cartwheel pattern. The plates then incubated in dark overnight at 37 °C. Then the plates were examined under UV light, and the minimum concentration of EtBr that resulted in fluorescence of bacterial colonies was recorded [18].

### Biofilm formation ability

The 96-well-plate based optical method was used. An overnight culture of *P. aeruginosa* in trypticase soy broth (Hi-Media, India) supplemented with 0.2% glucose was used to prepare an inoculum equivalent to a 0.5 McFarland standard. Ten µl of each isolate was then added to 190 µl of trypticase soy broth supplemented with 0.2% glucose into three wells of a 96-well flat-bottomed microtiter plate and incubated for 24 h at 37 °C. After incubation, the bacterial suspensions were carefully aspirated, and the wells were washed with 200 µl of phosphate-buffered saline (PBS) to remove planktonic cells. The wells were then decanted and air dried for 15 min. Subsequently, 200 µl of a 0.1% v/v crystal violet solution was added to each well and left to stand for 15 min. After staining, each well was washed with PBS to remove excess stain. Finally, the stained wells were solubilized with 200 µl of 33% v/v acetic acid, incubated at 37 °C for 15 min, and the optical density was measured at 630 nm. Negative control wells without inoculum served as controls. The results were recorded as the mean absorbance readings from triplicate wells [19]. Results were interpreted according to Sherif et al. (2021).

### Qualitative detection of pigment production

Cetrimide agar (Hi-Media, India) was used for qualitative analysis of *P. aeruginosa* pigment production. The method involved swabbing of overnight culture adjusted to OD of 0.5 MacFarland on the surface of the medium. The plates were then incubated for 24 h at 37 °C. After incubation, colonies that appeared blue green in colour were considered pyocyanin producers, while yellow colonies were considered pyoverdine producers [20].

### Assessment of swarming motility

The method described by Ha et al. (2014) [21] was employed. In brief, *P. aeruginosa* isolates were cultured in Lysogeny broth (LB) then incubated at 37 ^o^ C with shaking at 240 rpm. After that, 2.5 µL of the overnight cultures were inoculated on the surface of 0.5% agar plates. The inoculation was done in the centers of the plates with a pipette tip close to the surface of the media. The plates were then carefully transferred to the incubator and incubated upright on a flat non tilted surface at 30 °C with beaker full of water to preserve the humidity of the incubation chamber. Swarm zones were measured and classified into weak- moderate motility (zones less than 30 mm), high motility (zones more than 30 mm), and non-Swarmers (isolates that didn’t form tendrils).

### Detection of hemolysis

The ability of the isolates to produce hemolysis was evaluated following the protocol described by Buxton (2005) [22]. Blood agar plates were prepared from Tryptic Soya Agar (TSA) and supplemented with 5% sheep blood. Isolates were streaked on the surface in inoculum size of 1 × 10^8^ CFU/mL. After incubation, the plates were inspected for the presence of hemolysis zones.

**In-vivo Pathogenic Potential Assay**:

The non-mammalian host *Galleria mellonella* larvae was used as an infection model to test strain pathogenicity without the interference of other patients’ associated factors. It is important to mention that *G. mellonella* infection model was used by the authors in a recent publication [23].

This was done by subjecting groups of 10 larvae to be injected with 10 µL of bacterial suspension (10^4^ CFU/mL) to introduce a final cell count of approximately 10^2^ CFU/larva. The injection was done using a Hamilton syringe in the abdominal area in the last left pro-leg. One additional group of 10 larvae was injected with 10 µL of PBS and included in the study as negative control [24, 25].

### Statistical analysis

Bivariate analysis using Kruskal-Wallis test based on quantitative variables distribution by test of normality as well as Pearson’s Chi-square test compared different demographic and clinical parameters between categorical variables (resistotype, virulence factors). One-Way ANOVA followed by Post Hoc Tukey’s HSD was used to determine whether there are any statistically significant differences between the means of different groups. The mantel-Cox analysis was done to analyze the survival curves. All statistical tests were two-sided and judged at 0.05 significance level [26]. Statistical analysis was done using IBM SPSS statistics program [27] and R software packages [28] and GraphPad Prism 8.0.2 (GraphPad Software, USA).

## Results

### Bacterial identification and clinical history of the patients

A total of fifty- two non-duplicate clinical isolates were identified and confirmed as *P. aeruginosa* during the study period. The patient population consisted of equal male and female subjects with an age range of 1 to 66 years. The clinical isolates were collected from various hospital wards: medical, paediatric, and surgical wards with a total bed capacity of 350. All patients had an underlying malignancy, either solid tumor (SOT) of which 9 patients (23%) were neutropenic, or hematological malignancy (HM) of which 16 patients (80%) were neutropenic. Thirty-day mortality was recorded and defined as patients who died within 30 days of the infection. In our study, 14 patients (26.9%) died of which 10 of them (71.4%) were neutropenic. Demographic data and clinical history of patients are illustrated in (Table 1).

**Table 1:** Demographic data and clinical characteristics of study participants

Category		*n*.	%	
**Demographic Data**				
**Age group**	Pediatric	18	34.6%	
	Adult	34	65.4%	
**Gender**	Male	26	50.0%	
	Female	26	50.0%	
**Hospital Ward**	Medical	23	44.2%	
	Surgical	11	21.2%	
	Pediatric	18	34.6%	
**Clinical Data**				
**Diagnosis (Type of malignancy)**	HM	21	40.4%	
	SOT	31	59.6%	
**Neutropenic**	Negative	26	50.0%	
	Positive	26	50.0%	
**Clinical documented infection**	Surgical site infections	17	32.7%	
	Respiratory tract infections	20	38.5%	
	Urinary tract infections	15	28.8%	
**Previous antibiotic intake (one-month prior isolation)**	Monotherapy	39	75.0%	
	Combined therapy	13	25.0%	
**Outcome**	Dead	14	27%	
	Alive	38	73%	

*Clinical documented infection refers to the primary source of infection before bloodstream

RTI: respiratory tract infection, SSI: surgical site infection, UTI: urinary tract infection. HM: hematological malignancy, SOT: Solid tumors

### Antimicrobial susceptibility results

Most of the isolates (*n* = 35, 67.31%) were classified as MDR, while 16 isolates (30.77%) were XDR, and only one isolate (1.92%) displayed PDR phenotype which for statistical purposes was considered as XDR throughout the study. In addition, 19 isolates (36.5%) were recognized as DTR.

**Fig. 1:**
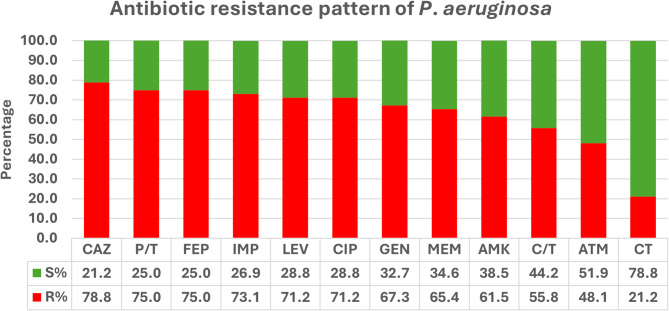
Antimicrobial resistance rates of *P. aeruginosa* clinical isolates. AMK: Amikacin; ATM: Aztreonam; C/T: Ceftolozane/Tazobactam; CAZ: Ceftazidime; CIP: Ciprofloxacin; CT: Colistin; FEP: Cefepime; GEN: Gentamicin; IMP: Imipenem; LEV: Levofloxacin; MEM: Meropenem; P/T: piperacillin/Tazobactam

Antimicrobial susceptibility and MIC results revealed that aztreonam and ceftolozane/tazobactam showed the lowest resistance levels among β-lactam antibiotics in which 25 isolates (48.1%) and 29 isolates (55.8%) were resistant respectively. Aminoglycosides showed lower resistance rates (Gentamicin: *n* = 35, 67.3% and amikacin: *n* = 32, 61.5%) compared to quinolones (*n* = 37, 71.2%). Colistin showed the lowest resistance rates in our collection (*n* = 11, 21.2%) (Fig. 1; Table 2).

**Table 2:** The MIC ranges of susceptible and non-susceptible isolates along with the MIC_**50**_**and MIC**_**90**_**of colistin**,** ceftolozane/tazobactam**,** imipenem**,** and Levofloxacin**

Number of Isolates													
Minimum Inhibitory concentration (µg/ml)	0.5	1	2	4	8	16	32	64	128	256	512	MIC_50_	MIC_90_
Colistin (CT)	0	0	6	35	6*	1*	4*	0	0	0	0	4	8
Imipenem (IMP)	0	3	4	7	1*	11*	6*	2*	2*	9*	7*	16	512
Levofloxacin (LEV)	3	12	2*	5*	8*	1*	6*	15*	0	0	0	8	64
Ceftolozane/Tazobactam (C/T)	0	24	0	0	1*	10*	2*	3*	12*	0	0	16	128

* Non-susceptible

A statistically significant results between the resistotype (MDR, XDR) and the hospital ward was observed in which XDR resistotype was higher in samples recovered from medical wards (*P* = 0.022). Moreover, a statistical significance was found between the resistotype and the clinical outcome in which MDR isolates were associated with lower mortality than that observed in case of XDR (*P* = 0.0429) (Table 3).

**Table 3:** Association between patient characteristics and bacterial resistotypes (MDR/ XDR)

Patients’ Data				Resistotype				*P*-value
Demographic data		Total		MDR		XDR		
		*n*.	%	*n*.	%	*n*.	%	
Age group	Paediatric	18	34.6%	14	40.0%	4	23.5%	0.242
	Adult	34	65.4%	21	60.0%	13	76.5%	
Gender	Male	26	50.0%	18	51.4%	8	47.1%	0.768
	Female	26	50.0%	17	48.6%	9	52.9%	
Hospital Ward	Medical	23	44.2%	11^a^	31.4%	12 ^b^	70.6%	0.022*
	Surgical	11	21.2%	10 ^a^	28.6%	1 a	5.9%	
	Paediatric	18	34.6%	14 ^a^	40.0%	4 a	23.5%	
Clinical history								
Diagnosis	HM	21	40.4%	16	45.7%	5	29.4%	0.261
	SOT	31	59.6%	19	54.3%	12	70.6%	
Neutropenic	Negative	26	50.0%	19	54.3%	7	41.2%	0.375
	Positive	26	50.0%	16	45.7%	10	58.8%	
Previous clinical documented infections (CDI)	SSI	17	32.7%	10	28.6%	7	41.2%	0.427
	RTI	20	38.5%	13	37.1%	7	41.2%	
	UTI	15	28.8%	12	34.3%	3	17.6%	
Previous antibiotic intake (one-month prior isolation)	Monotherapy	39	75.0%	26	74.3%	13	76.5%	FEp1
	Combined therapy	13	25.0%	9	25.7%	4	23.5%	
Outcome	Dead	14	27%	6 ^a^	17.14%	8 ^ab^	47.06%	0.0429*
	Alive	38	73%	29 ^b^	82.86%	9 ^ab^	52.94%	

Chi-square significance, different superscript letters denote significant pairwise comparison. * Significant results ≤ 0.05. HM: hematological malignancy, SOT: solid tumors, SSI: surgical site infection, RTI: respiratory tract infection, UTI: urinary tract infection

**Fig. 2:**
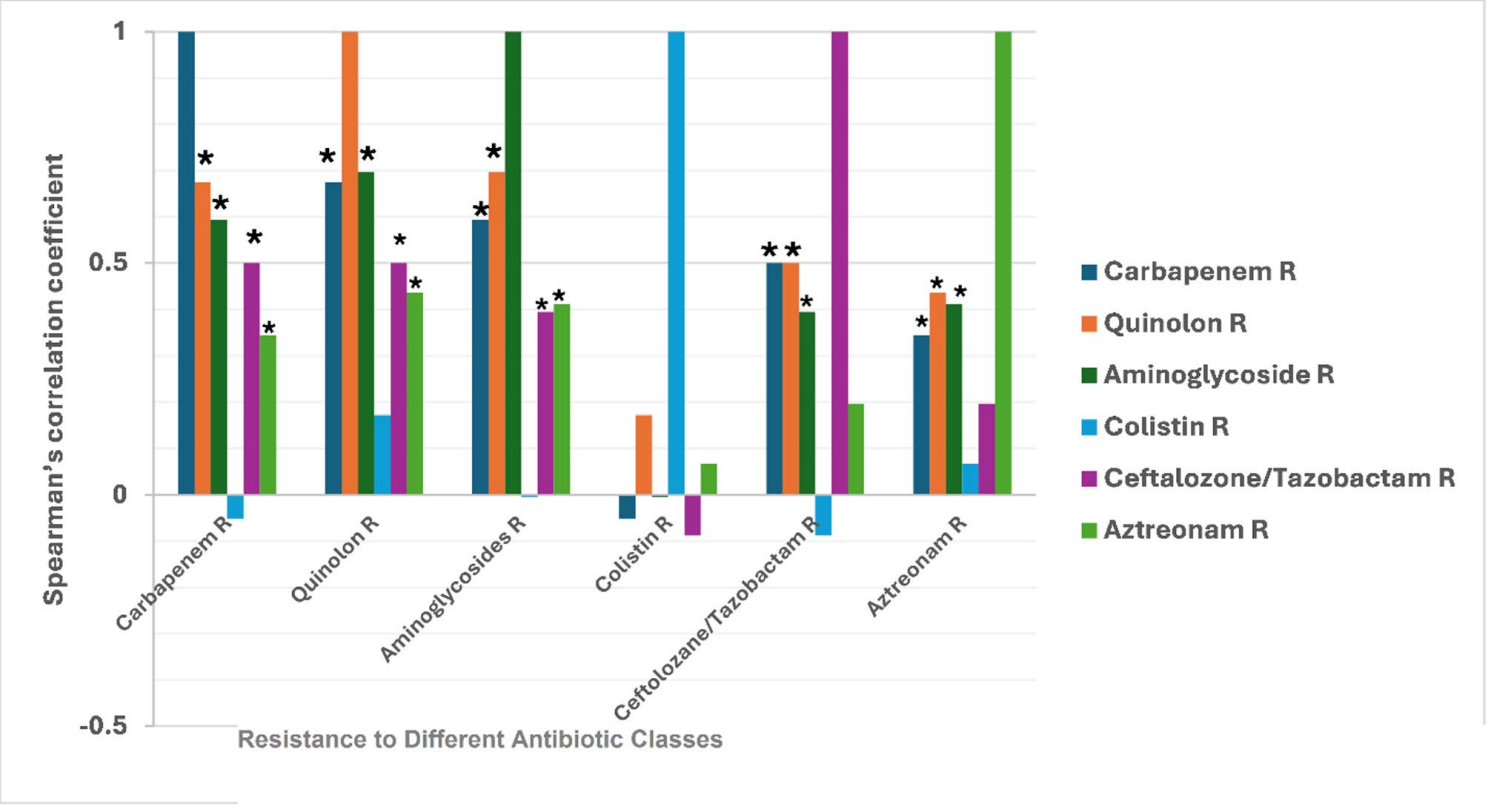
Spearman’s correlation coefficient illustrates the co-existence of resistance between different antibiotic classes. * Significant correlation. This figure illustrates the strength of association between resistance to various antibiotic classes in *P. aeruginosa* isolates, as determined by Spearman’s correlation analysis. The y-axis represents Spearman’s correlation coefficient (r), ranging from − 0.5 to 1, where values closer to 1 indicate a strong positive correlation, suggesting co-resistance patterns. Each group on the x-axis denotes resistance to a specific antibiotic class, and within each group, the colored bars represent the correlation of that resistance with other antibiotic classes. The strongest positive correlations were observed between: Carbapenem resistance and quinolone resistance, carbapenem resistance and aminoglycoside resistance, and quinolone resistance and aminoglycoside resistance, indicating potential multidrug resistance clusters

Statistically significant results were highly observed in the co-existence of resistance among the key antibiotic classes in which a strong positive correlation was detected between carbapenem/quinolones (92.5%, rs = 0.675, *p*-value < 0.00001); and aminoglycosides /quinolones resistance (90%, rs = 0.696, *p*-value < 0.00001). A moderate positive correlation was detected between carbapenem/aminoglycosides (87.5%, rs = 0.594, *p*-value < 0.00001); carbapenem/ ceftolozane-tazobactam (67.5%, rs = 0.5, *p*-value < 0.00001); ceftolozane–tazobactam/ quinolones (67.5%, rs = 0.5, *p*-value < 0.00001); aztreonam/quinolones (60%, rs = 0.436, *p*-value 0.001); and aztreonam/ aminoglycosides (60.5%, rs = 0.411, *p*-value 0.003). Weak positive correlation was detected between ceftolozane–tazobactam/ aminoglycosides (67.5%, rs = 0.395, *p*-value 0.004) and carbapenem/aztreonam (57.5%, rs = 0.344, *p*-value 0.012). On the contrary, no statistical significance was detected between colistin and other antibiotics (Fig. 2).

**Fig. 3:**
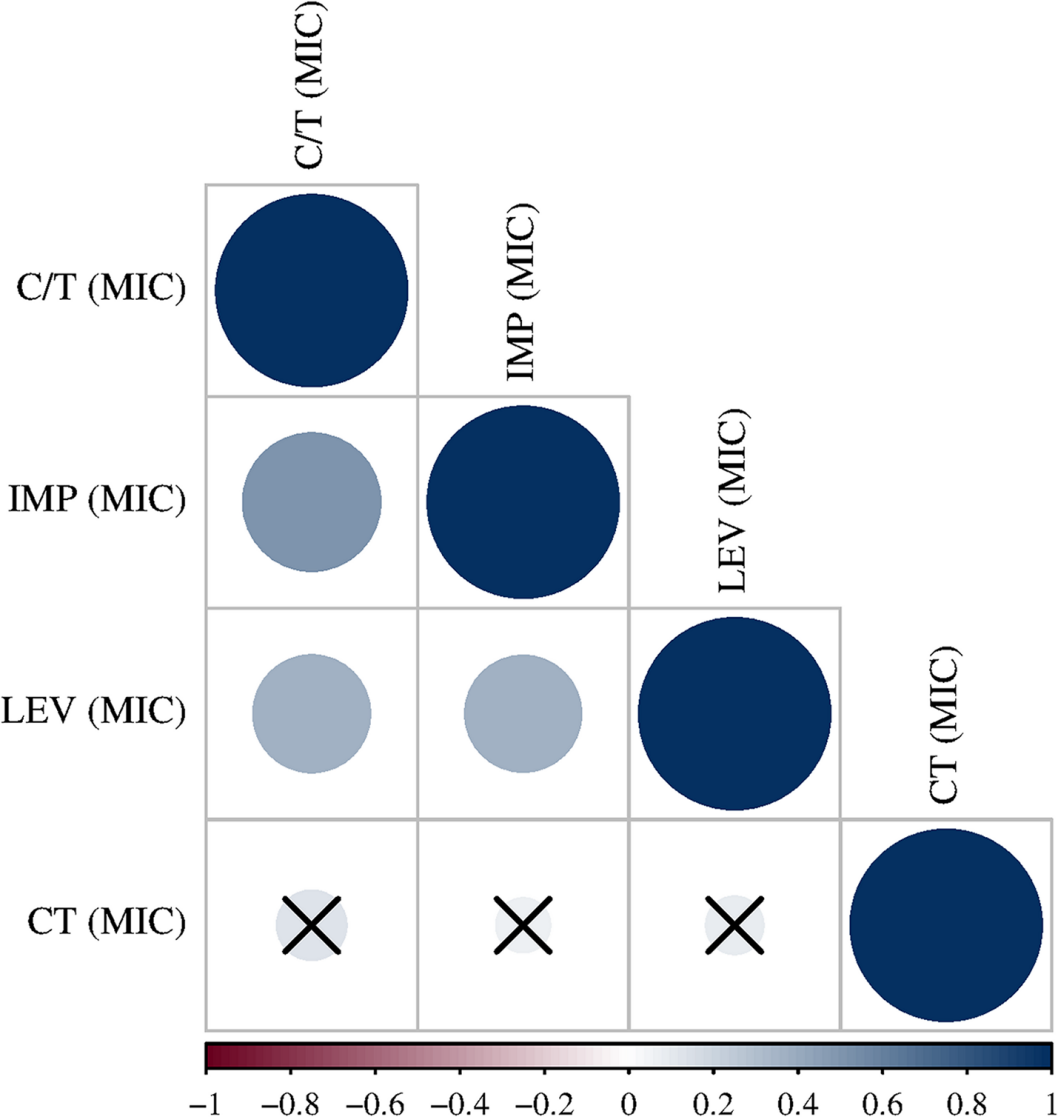
Correlogram representing Spearman’s correlation (rs) between MICs of Ceftolozane/Tazobactam (C/T), Imipenem (IMP), Levofloxacin (LEV), and Colistin (CT). × Denote insignificant results

A statistically significant moderate positive correlation was found between the MIC of IMP with both C/T (rs = 0.486, *p*-value 0.0002) & LEV (rs = 0.454, *p*-value 0.0007), whereas a weak positive correlation was found between the MICs of LEV and C/T (rs = 0.33, *p*-value 0.015) (Fig. 3).

### Phenotypic detection of MβL & carbapenemase resistance genes

The combined disc test for phenotypic detection of MβL production and the qualitative detection of carbapenemases that was performed on all isolates showed that 34 (65.4%) isolates were MβLand/or carbapenemase producers in which 32 (94.1%) of them harboring *bla*_*NDM−like*_ while only 2 (5.9%) isolates carried *bla*_*KPC−like*_. None of the other tested carbapenemases were detected in our collection. Among the MβL and/or carbapenemase producing isolates there were isolates retained the susceptibility towards carbapenems and other β-lactams (Aztreonam, Ceftolozane/Tazobactam, Piperacillin/Tazobactam, Cefepime, Ceftazidime, and Amoxicillin/clavulanic acid). It is interesting that susceptibility to aztreonam and ceftolozane/tazobactam was retained by 41.2% and 29.4% of the MβL and carbapenemase-producing isolates, respectively. A statistical significance between the presence of MβL/Carbapenemases and β-lactam resistance was detected in piperacillin/tazobactam, ceftazidime, cefepime, meropenem, and imipenem with *p*-value < 0.001 for all. The absence of these genes was significantly associated with susceptibility to both Aztreonam (*p*-value 0.003) and ceftolozane/tazobactam (*p*-value 0.009).

### Efflux pump activity of MDR/XDR *P. aeruginosa* clinical isolates and its effect on MIC

**Fig. 4:**
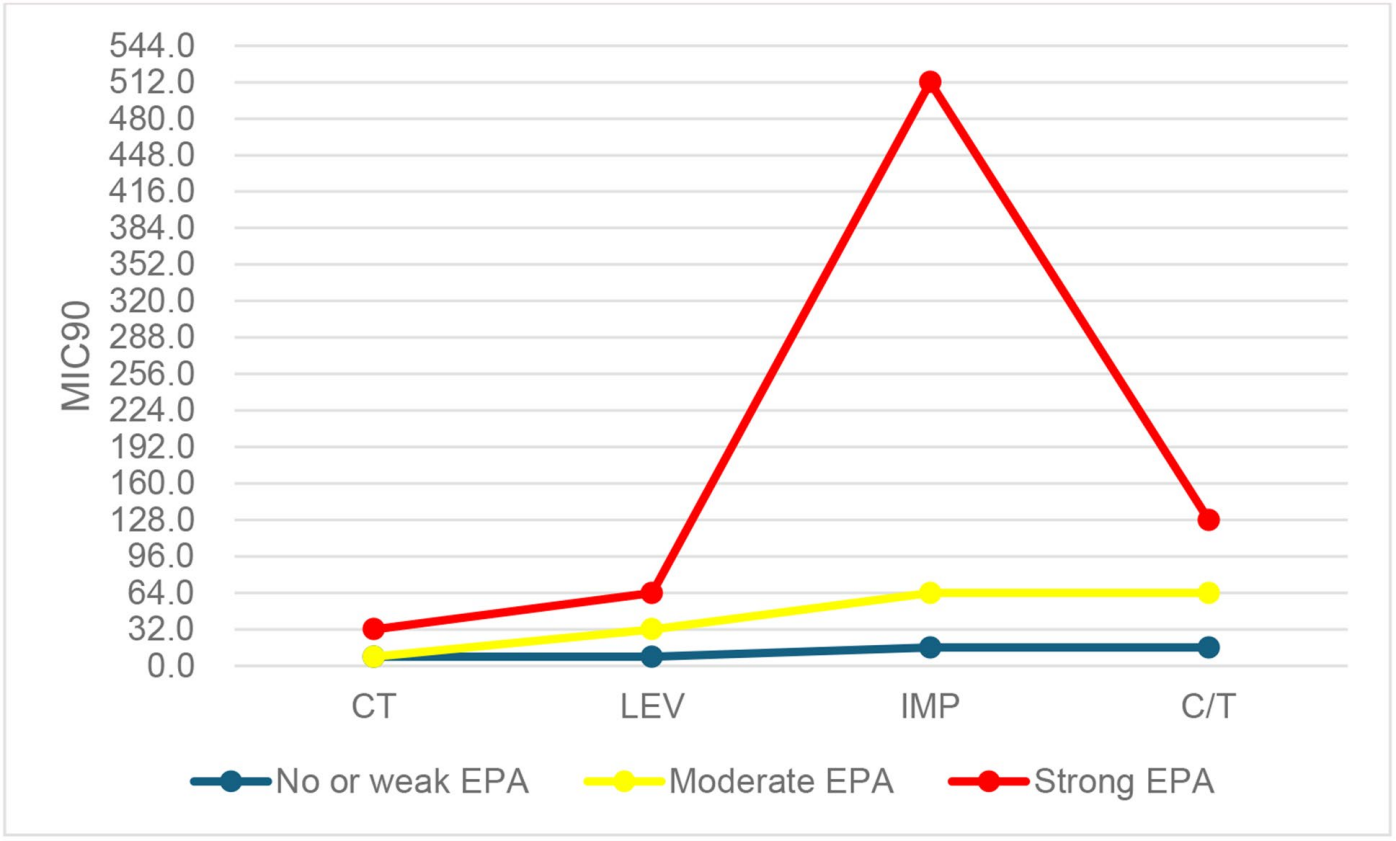
The effect of the strength of the EPA on the MIC_90_ values for (C/T: ceftolozane/ tazobactam, CT: colistin, IMP: imipenem, LEV: levofloxacin)

Twelve isolates (23%) were considered to have no or weak EPA, 10 isolates (19%) have moderate EPA, whereas 30 isolates (58%) have strong EPA. The effect of the intensity of EPA on the MIC _90_ of IMP, CT, C/T, and LEV was assessed. Results revealed that isolates with no or weak EPA showed MIC_90_ ≤ 16 µg/mL for the 4 antibiotics. Higher MIC_90_ values were observed among isolates with strong EPA in which the MIC_90_ for colistin, levofloxacin, ceftolozane/tazobactam, and imipenem were 32, 64, 128, 512 µg/mL respectively. Our results showed a significant association between the MIC_90_ and the strength of EPA with *p*-value < 0.0001 (Fig. 4).

### Assessment of other virulence determinants

The biofilm forming ability results revealed that all the isolates were biofilm producers in which 4 isolates (7.7%) were weak biofilm formers, 28 isolates (53.8%) were moderate, and 20 isolates (38.5%) were strong biofilm formers.

Pigment production results showed that 41 isolates (79%) were able to produce pigment in which 22 isolates (42.3%) were pyoverdine producers whereas 19 isolates (36.5%) isolates were pyocyanin producers.

Regarding the swarming motility, results showed that 38 isolates (73%) exhibited swarming motility. Among these 15 (39.5%) were highly motile whereas the rest show moderate to weak motility.

**Fig. 5:**
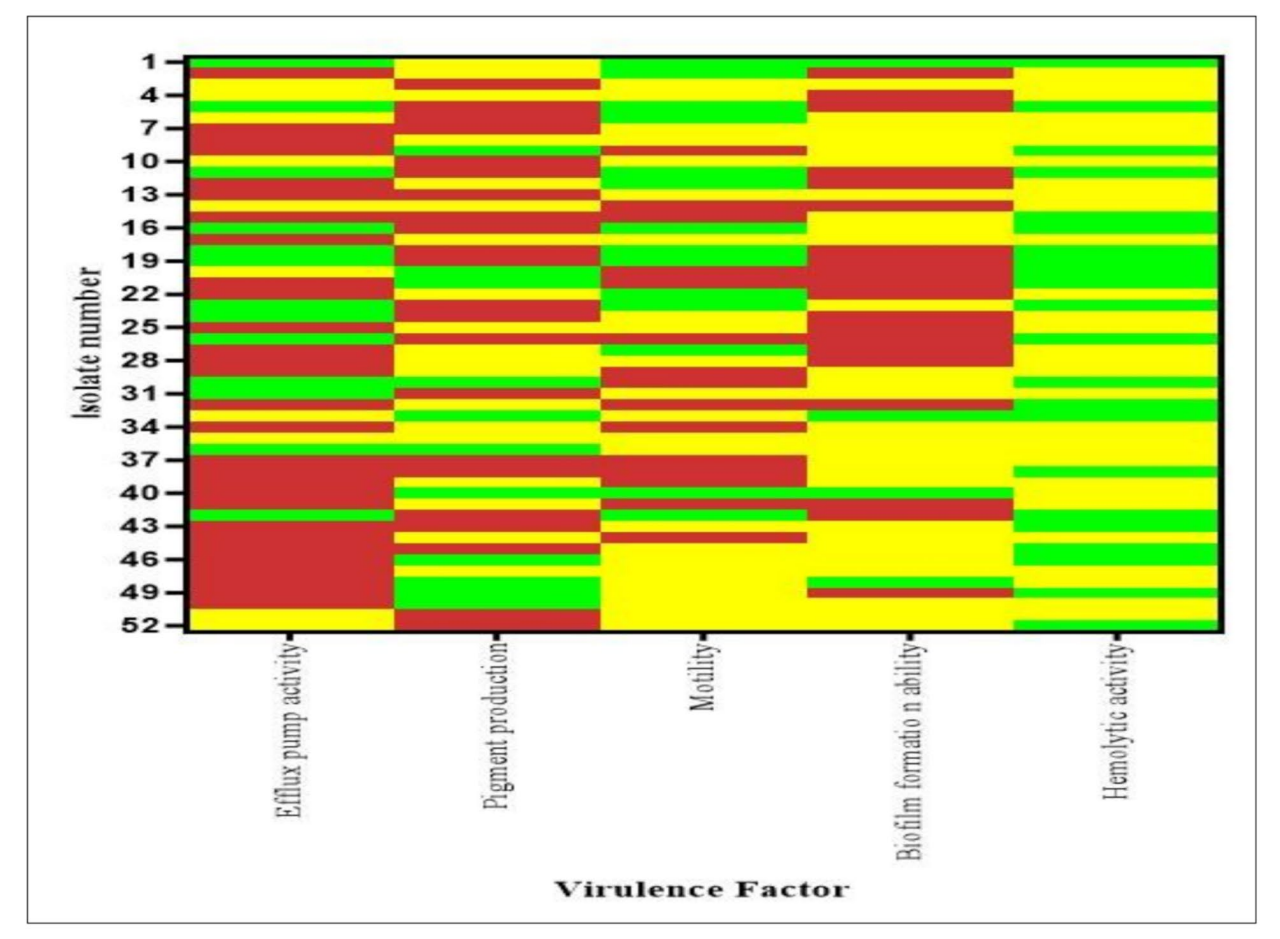
Overview of the *P. aeruginosa* clinical isolates virulence traits. The heatmap is color coded. Efflux Pump Activity: green = no or weak, yellow = moderate, red = strong. Pigment production: green = no production, yellow = pyocyanin production, red = pyoverdine production. Motility: green = no motility, yellow = weak-moderate, red = strong. Biofilm: green = weak, yellow = moderate, red = strong. Hemolytic activity: green = negative, yellow = positive

The hemolytic activity assessment revealed that 30 isolates (58%) were able to hemolyze blood whereas 22 isolates (42%) didn’t display hemolytic phenotype (Fig. 5).

ANOVA, followed by post-hoc Tukey HSD test showed a significant association between the different virulence determinants and the number of non-susceptible isolates toward each antibiotic. Significant association between non-susceptibility towards the used antibiotics were found to be higher in case of strong EPA (*p* < 0.00001) and moderate/strong biofilm producers (*p* < 0.00001). Additionally, pyoverdine production was found to be highly associated with non-susceptibility to antibiotics than pyocyanin production (*p* = 0.00024). It is noteworthy to mention that weak-moderate swarmers were highly associated with non-susceptibility toward the tested antibiotics than strong and non swarmers with *p*-value = 0.00125 and 00432 respectively. Hemolytic activity was also associated with non-susceptibility towards antibiotics (*p* = 0.000686).

### Virulence- virulence relation and their production level associations in MDR/XDR *P. aeruginosa* clinical isolates

**Fig. 6:**
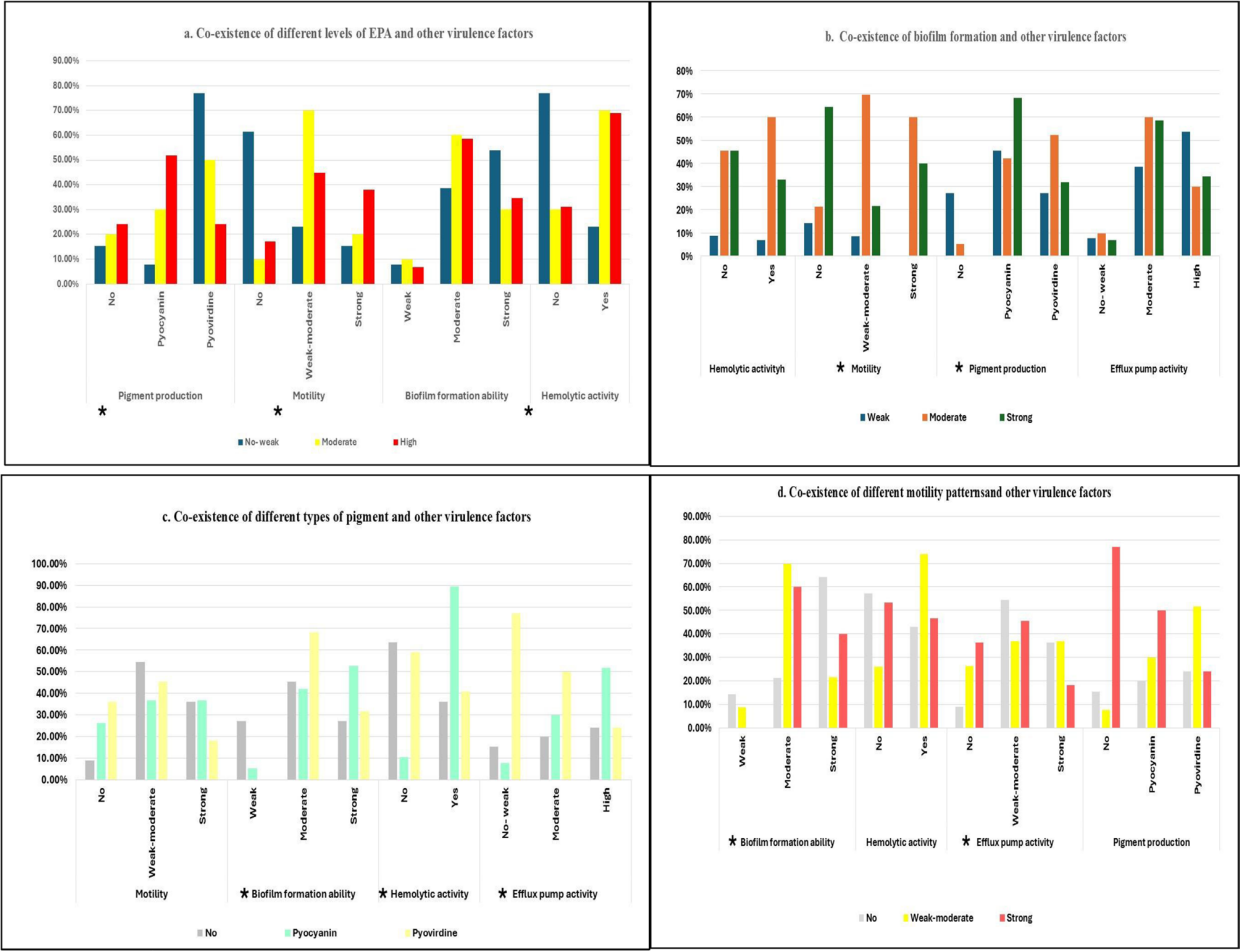
The interrelationships between different virulence factors in *P.aeruginosa* isolates **a.** Efflux pump activity, this panel categorizes EPA into three levels—no/weak (blue), moderate (yellow), and high (red)—and compares their associations with pigment production (pyocyanin, pyoverdine), motility, biofilm formation ability, and hemolytic activity. **b.** Biofilm formation, this panel explains how weak, moderate, and strong biofilm-forming abilities relate to hemolytic activity, motility, pigment production, and EPA. **c.** Pigment production, isolates are grouped by the type of pigment they produce, and their associations with motility, biofilm formation, hemolysis, and EPA are shown. **d**. Swarming motility, motility is categorized as weak, moderate, or strong, and its relationship to other factors is shown. * Presence of significant correlation

The association of different studied virulence factors were highly observed in our study. A significant association was detected between the intensity of EPA and the pigment production (*p*-value 0.02), low EPA was associated with absence or low levels of motility (*p*-value 0.011). Hemolytic activity was significantly associated with absence or low EPA (*p*-value 0.014). Absence of pyoverdine production was associated with weak biofilm formation (*p*-value 0.028). Whereas Pyocyanin production was directly related to the hemolytic activity (*p*-value 0.002). Moreover, a significant association was detected between the absence of swarming motility and strong biofilm formation (*p*-value 0.036) (Fig. 6).

### In-vivo pathogenic assay using *G. mellonella* (non- mammalian model)

**Fig. 7:**
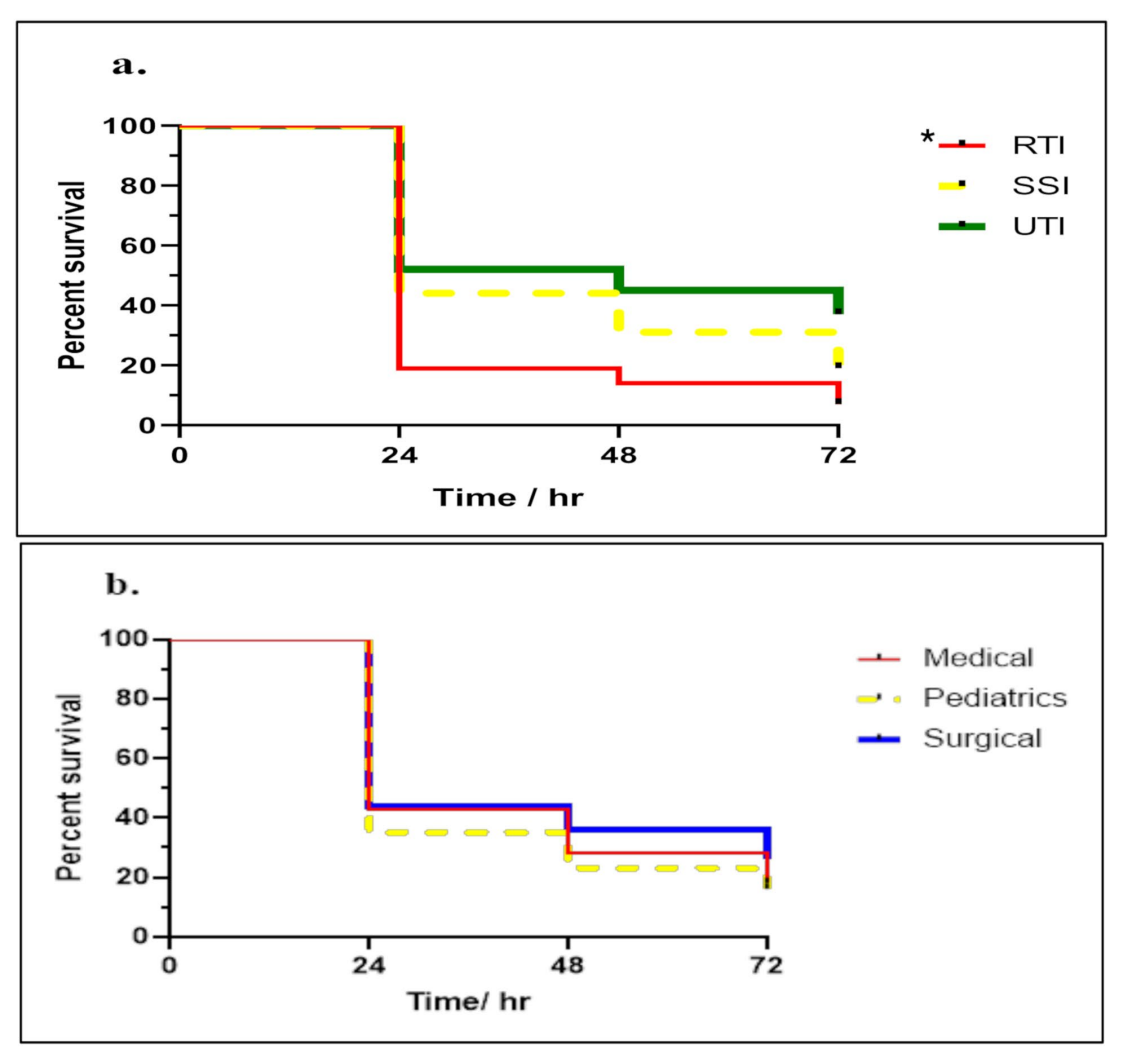
Survival curve of *G.mellonella* larvae over 72 h. (**a**) Infected with *P. aeruginosa* isolates from different sites of infections RTIs = respiratory tract infections, SSIs = surgical site infections, UTIs = urinary tract infections. (**b**) Infected with *P. aeruginosa* isolates from different hospital wards. * Significant correlation

*P. aeruginosa* isolates were classified into 3 groups according to the site of infection (Respiratory tract infections, surgical site infections, and urinary tract infections).

and another classification was done according to the ward into 3 groups (Medical, Paediatrics, and surgical). Results showed that high lethality to *G. mellonella* were observed in case of RTIs isolates (93.5%) followed by SSIs (82%) then UTIs (64%) with observed statistically significant relation with *p*-value = 0.001. Regarding the classification according to wards, results revealed that paediatric ward showed the highest lethality (84%) followed by medical (82%) and surgical ward (74%) with no statistically significant relation observed (*P* = 0.1609) (Fig. 7).

## Discussion

This study examined 52 *Pseudomonas aeruginosa* clinical isolates obtained from blood samples of hospitalized cancer patients. By evaluating both the demographic and clinical characteristics of patients, along with antimicrobial resistance (AMR) profiles of the isolates, we gained valuable insights into the epidemiology and resistance dynamics of *P. aeruginosa* within this high-risk population.

The patient cohort was evenly split by sex, with a considerable proportion (34.6%) being pediatric patients. The age range spanned from 1 to 66 years, reflecting that *P. aeruginosa* bloodstream infections (BSIs) in cancer patients can occur across all age groups, although the majority of cases (65.4%) were adults. This aligns with previous reports indicating that adult cancer patients are more frequently affected by *P. aeruginosa* BSIs [3, 29].

Solid tumors accounted for 62% of the underlying malignancies, while 38% were hematological cancers. A notable 80% of patients with hematologic malignancies were neutropenic, underscoring their increased susceptibility to severe infections. Neutropenia, a key risk factor for infections due to compromised immunity, is well-documented to contribute significantly to infection-related morbidity and mortality.

In this context, a study from China investigating 293 hospitalized adults with acute leukemia and *P. aeruginosa* BSIs found that multidrug-resistant (MDR) infections markedly elevated 30-day mortality (28.9% in MDR cases vs. 5.5% in non-MDR cases) [29]. Similarly, in our study, we observed a high crude 30-day mortality rate of 26.9%, with mortality significantly higher among patients infected with extensively drug-resistant (XDR) isolates compared to MDR isolates. These findings align with previous research reporting 30-day mortality rates between 16.1% and 32.5%, often associated with higher resistance levels [3, 30, 31].

A majority of patients (71%) had received antibiotic monotherapy in the month preceding sample collection, potentially contributing to the emergence of resistant strains via selection pressure. The diversity of infection sites primarily the respiratory tract (38.4%), surgical sites (32.7%), and urinary tract (28.9%) highlights the opportunistic nature of *P. aeruginosa* in cancer patients.

The antimicrobial resistance patterns observed in our study are particularly concerning. Resistance rates to commonly used antibiotics including beta-lactams, quinolones, aminoglycosides, and carbapenems—were exceptionally high. All isolates displayed either MDR (67.3%) or XDR (30.7%) phenotypes, with one pan-drug resistant (PDR) isolate detected, severely limiting treatment options. These high resistance rates are likely influenced by our study setting: a major tertiary cancer referral hospital in Egypt that treats critically ill, immunocompromised patients frequently exposed to antibiotics. The patient population is predominantly immunocompromised due to underlying malignancies and intensive treatments such as chemotherapy, which increases susceptibility to infections and often requires frequent or prolonged antibiotic treatment. These factors, combined with repeated healthcare exposures and potential prior colonization, contribute significantly to the high prevalence of multidrug-resistant organisms observed in our collection.

Our resistance rates surpass those reported globally [3, 31] and are also higher than figures from some local Egyptian studies [8, 32], which reported resistance levels of 68–79.8%. However, other Egyptian reports have documented MDR/XDR prevalence as high as 100%, in line with our findings [33].

Despite the widespread resistance, our isolates showed comparatively higher susceptibility to colistin, aztreonam, and ceftolozane/tazobactam. Colistin had the highest susceptibility rate (78.8%), consistent with prior Egyptian studies reporting 79.4–100% susceptibility [32, 34–36]. Aztreonam was the second most effective (51.9%), although this was lower than global and earlier national estimates [3, 30, 32, 36], likely due to the presence of β-lactamases (e.g., AmpC or ESBLs) capable of hydrolyzing aztreonam. The coexistence of multiple resistance mechanisms in *P. aeruginosa* has been increasingly reported, particularly in high-risk settings with intensive antibiotic use such as our tertiary cancer center.

Ceftolozane/tazobactam susceptibility was 44.2%, which is lower than reported both internationally [37, 38] and within Egypt [39]. This may be partly attributed to the hospital’s frequent use of meropenem as empirical therapy—a factor associated with ceftolozane/tazobactam resistance [40]. The high prevalence of *bla*_*NDM−1*_ gene (found in most isolates) likely also contributes to the reduced effectiveness of this novel combination. Notably, 83% of ceftolozane/tazobactam-resistant isolates were also meropenem-resistant.

Our data revealed a 65.3% prevalence of metallo-β-lactamase (MBL)-producing *P. aeruginosa*, with other Egyptian studies reporting variable rates [32, 41, 42] and lower rates globally [43, 44]. Among detected carbapenemase genes, *bla*_*NDM−1*_ was most common, corroborating its endemic nature in our hospital, as previously reported [45–48]. Interestingly, some MBL-producing isolates retained susceptibility to aztreonam (41.2%), suggesting that in MBL-endemic settings, aztreonam—due to its resistance to hydrolysis by class B β-lactamases—may remain a viable treatment option following colistin.

High rates of cross-resistance were observed among key antibiotic classes, including quinolones, aminoglycosides, aztreonam, and ceftolozane/tazobactam. However, colistin resistance showed no statistical correlation with other classes. These findings align with previous studies [49] and are often attributed to the presence of efflux pumps such as MexAB-OprM and MexXY, which mediate resistance to multiple drug classes [50–52]. Our study demonstrated a direct association between efflux pump activity (EPA) and MIC_90_ values, where isolates with higher EPA exhibited reduced susceptibility. This poses significant treatment challenges, including increased dosage needs, prolonged therapy, and greater risk of adverse outcomes and resistance amplification. Efflux systems also contribute to bacterial virulence and stress responses [50].

All isolates in this study were capable of biofilm formation, with a predominance of moderate/strong biofilm producers, consistent with previous reports from Egypt [8, 53]. Stronger biofilm producers showed reduced susceptibility to antibiotics, in line with literature suggesting that biofilms impair antibiotic penetration and alter bacterial metabolism [6, 8, 53, 54]. In contrast, some studies found no such correlation, likely due to differences in how biofilm formation was measured [18, 55].

Pyoverdine was the most commonly produced pigment, followed by pyocyanin, mirroring earlier findings [8]. Pyoverdine production was associated with lower antibiotic susceptibility and stronger biofilm formation, as supported by literature [18, 56–58]. Swarming motility was observed in 73% of isolates, with a significant association between strong swarming and urinary tract infections, consistent with studies linking motility to uropathogenicity [59, 60].

The relationship between swarming and resistance is debated. In our study, weak to moderate swarmers were more resistant, while strong swarmers showed less resistance, suggesting a potential trade-off between motility and resistance—possibly due to fitness cost, as proposed in other studies [57, 58, 61–66].

We also found interplay among virulence traits. EPA correlated positively with pyocyanin production and negatively with motility echoing reports that efflux systems influence both pigment production and flagellar function [67–69]. Similarly, pyoverdine production was linked to strong biofilm formation, and swarming motility inversely correlated with biofilm strength, in agreement with several large-cohort studies [58, 70], though some studies report the opposite [57, 71].

Pathogenic potential assessed using the *Galleria mellonella* model showed higher lethality in isolates from respiratory infections, in line with studies showing that respiratory-origin strains often produce higher levels of exoU/exoS toxins key virulence factors linked to poor outcomes [72–79].

The limitations of this study include the single-center design, the limited and descriptive nature of the clinical data, the grouping of heterogeneous patient populations without detailed stratification, and the broad categorization of infection types. We acknowledge that the primary focus of the study on the microbiological characterization of isolates. Future studies should incorporate detailed patient-level data to better differentiate infection types and correlate microbial traits with clinical outcomes and risk factors.

In conclusion, our findings highlight the significant burden of *P. aeruginosa* infections in immunocompromised cancer patients, characterized by high resistance rates, especially to frontline antibiotics. The predominance of MDR and XDR isolates, with frequent detection of *bla*_*NDM−1*_, underscores the urgent need for alternative therapeutic options and robust antimicrobial stewardship.

Virulence traits such as biofilm formation, pigment production, motility, and EPA were widespread and often interrelated, complicating treatment strategies. Biofilm production was particularly linked to reduced antibiotic efficacy, and complex associations were observed between virulence factors and resistance mechanisms.

The *Galleria mellonella* infection model confirmed the high pathogenic potential of isolates, especially those from respiratory tract infections, likely due to the presence of exoU/exoS toxins.

These results call for enhanced infection control, continuous resistance surveillance, and novel treatment strategies to manage *P. aeruginosa* infections in high-risk populations.

## Data Availability

Data is provided within the manuscript.
